# A Comprehensive Approach for Designing Low Carbon Wood Bio-Concretes

**DOI:** 10.3390/ma17112742

**Published:** 2024-06-04

**Authors:** M’hamed Y. R. da Gloria, Lucas R. Caldas, Joaquim A. O. Barros, Romildo D. Toledo Filho

**Affiliations:** 1 Department of Civil Engineering, COPPE, Universidade Federal do Rio de Janeiro, Cidade Universitária, Rio de Janeiro CEP 21941-972, Brazil; yassin@numats.coc.ufrj.br (M.Y.R.d.G.); lrc@numats.coc.ufrj.br (L.R.C.); 2 ISISE, ARISE, Civil Engineering Department, Universidade do Minho, Campus de Azurém, 4800-058 Guimarães, Portugal; barros@civil.uminho.pt

**Keywords:** wood bio-concretes, mix design, supplementary cementitious materials, GHG emissions, bio-based building materials

## Abstract

This paper presents a method for designing low carbon bio-based building materials, also named bio-concretes, produced with wood wastes in shavings form (WS) and cementitious pastes. As the aggregates phase of bio-concretes is composed of plant-based particles, known as porous and high water-absorbing materials, the bio-concretes cannot be designed by using the traditional design rules used for conventional mortar or concrete. Then, the method used in the current paper is an adaptation of a previous one that has been developed in a recent paper where bio-concretes were produced with a cement matrix, three types of bio-aggregates, and a proposal of a design abacus. However, when that abacus is used for designing WBC with low cement content in the matrix, the target compressive strength is not reached. In the present paper, the method is extended to low cement content matrix (up to 70% of cement substitution) and also considering the greenhouse gas (GHG) emission of the WBC. To obtain data for proposing a new design abacus, an experimental program was carried out by producing nine workable WBCs, varying wood volumetric fractions (40–45–50%), and water-to-binder ratios. The bio-concretes produced presented adequate consistency, lightness (density between 715 and 1207 kg/m^3^), and compressive strength ranging from 0.64 to 12.27 MPa. In addition, the GHG emissions of the WBC were analysed through the Life Cycle Assessment methodology. From the relationships obtained between density, compressive strength, water-to-binder ratio, cement consumption, and GHG emissions of the WBC, calibration constants were proposed for developing the updated and more complete abacus regarding an integrated mix design methodology.

## 1. Introduction

In a previous paper, da Gloria et al. [1] proposed a method for designing workable wood, bamboo, and rice husk bio-concretes, produced with a cement matrix. The dosage methodology consisted successively on: (i) evaluation of the chemical compatibility between bio-aggregates (BA) and cement in order to define the BA treatment; (ii) determination of the compensating water, based on the water absorbed by the BA during the bio-concretes production; (iii) definition of the fresh bio-concretes spreading range (275 ± 30 mm); (iv) selection of a volumetric fraction of bio-aggregates (45–52.5%); and (v) determination of the water-to-cement ratio (0.40–0.50) that allows for the framing of fresh bio-concrete in the pre-defined spreading range. The compensating water is an additional water introduced during the mix process to guaranty that the BA would be saturated and would not absorb the free water required for cement hydration. The selected spreading aimed to obtain bio-concretes that dispensed the necessity of the pressing process during the casting [2,3,4,5]. After achieving workable bio-concretes, an experimental campaign was conducted to access the influence of the BA content and the water-to-cement ratio on the density and compressive strength of the bio-concretes. According to each bio-aggregate adopted, the hardened state properties of the corresponding bio-concrete have varied. For example, the wood bio-concretes (WBC) and the bamboo bio-concretes (BBC) compressive strength ranged from 3.68 to 8.81 MPa and 2.52 to 4.57 MPa, respectively. In terms of density, WBC presented density from 820 to 950 kg/m^3^, while BBC density ranged from 620 to 800 kg/m^3^. Based on the obtained results, relationships between dosage parameters and hardened bio-concretes properties were derived for developing a rational mix design diagram, extensible to others types of bio-concretes.

Before that method, only empirical dosage methods based on the cement-to-BA mass ratio were used in the literature [2,5,6] and the industrial field [7,8] to produce the bio-concretes. When the bio-concrete is manufactured as particleboard, the cement-to-wood mass ratio varies between 2 and 3 (equivalent to a wood volumetric fraction between 45 and 55% in most of the cases) and the water-to-cement (*w*/*c*) ratio ranges from 0.15 to 0.17. Such low *w*/*c* ratio is due to the high pressure applied during the moulding process for the fresh bio-concrete compaction in the formwork. From this casting process, we obtained panels of density, compressive strength, and Young’s modulus 1250 kg/m^3^, 15 MPa and 4.5 GPa, respectively. The WBC produced are nonstructural materials, usually applied as cladding panels for facades, partition walls, cladding of interior walls, roof supports, and finishing elements or ceiling cladding elements.

The authors in the previous paper [1] also emphasized that even if the goal of the study was to propose a new bio-concrete mix design method, a matrix containing only cement was not the most sustainable choice in terms of environmental impact. For that reason, they highlighted the importance of introducing supplementary cementitious materials (SCM) in further bio-concretes matrices.

The environmental impacts and benefits of the SCM employment for bio-concrete manufacturing was evaluated by Caldas et al. [9]. The authors produced seven WBC made with matrix containing cement partially replaced (40% to 50%, by mass) with different combinations of fly ash (FA) and metakaolin (MK). They also performed both a compressive test and a cradle-to-gate Life Cycle Assessment (LCA). On the one hand, they observed that such a replacement can improve the mechanical strength of the WBC up to 30%. On the other hand, 50% of cement replacement can reduce the global warming potential (GWP) up to 39 kgCO_2_-eq/m^3^. Lima et al. [10,11,12] also produced WBC, substituting up to 60% of cement by FA, MK, rice husk ash, and blast furnace slag, and achieved compressive strength between 80 and 110% of the values presented by the WBC with only the cement matrix.

In this context, the present research proposes an improvement of the wood bio-concretes mix design methods by taking into account the target density, compressive strength, volumetric fraction, but also the greenhouse gas (GHG) emissions. The GHG emissions were assessed through LCA tools applied on WBC developed and characterized in an experimental campaign. The WBC matrix was composed of cement partially replaced by a combination of metakaolin and fly ash as a strategy to reduce carbon emissions.

## 2. Materials and Methods

### 2.1. Materials

The wood shavings (WS) were obtained from the carpentry Madeirama in the city of Rio de Janeiro (Brazil) and consisted of a mix of four species wastes (*Manilkara salzmanni*, *Hymenolobium petraeum*, *Cedrela fissilis*, and *Erisma uncinatum Warm*) randomly collected according to the daily availability. Based on da Gloria and Toledo [5], only the particles retained on a 1.18 mm sieve were used to produce the bio-concretes. Next, the WS were homogenised by using the elongated pile as a sampling method.

**Figure 1:**
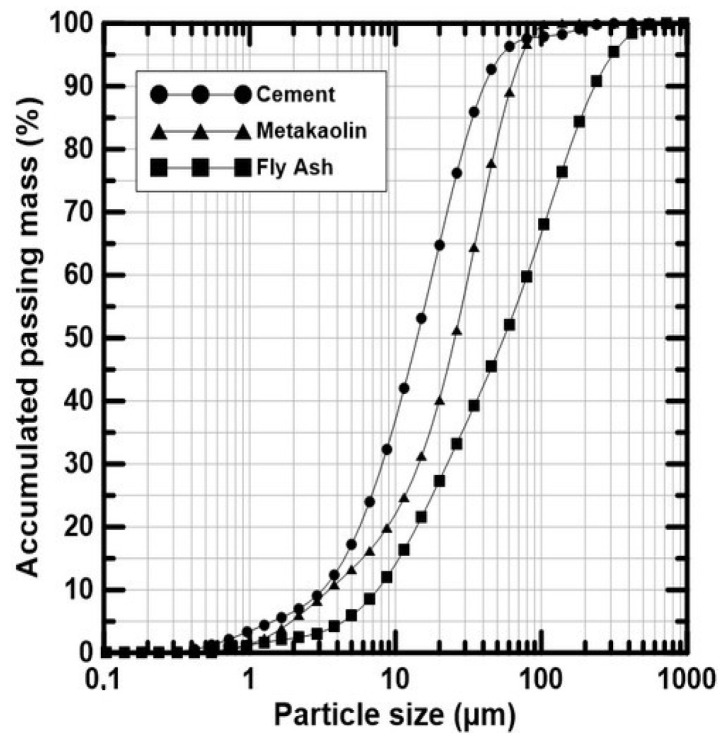
Granulometric curves of CEM, MK, and FA.

The binder was composed of a ternary combination (by mass) of Brazilian Portland cement labelled CP V-ARI (CEM) from Lafarge-Holcim (Rio de Janeiro, RJ, Brazil), metakaolin (MK) supplied by Metacaulim do Brasil (Jundia, SP, Brazil) and fly ash (FA) from Pozo Fly Comércio de Cinzas Lima LTDA (Capivari de Baixo, SC, Brazil), in proportions of 30%, 30%, and 40%, based on Caldas et al. [9] developments. The supplementary materials (FA and MK) were used for reducing the cement content and obtain a cementitious paste free of hydroxide calcium after 28 days. The chemical composition and the granulometric curves of the cementitious materials are exposed in Table 1 and Figure 1, respectively. To guarantee a demoulding after 24 h, 3% of calcium chloride (based on the binder mass) was used to accelerate the cementitious paste setting, as recommended in the literature [2,6,12,13].

### 2.2. Wood Shavings Treatment

The WS were treated before the WBC production in order to improve their chemical compatibility with the cement. According to Alberto et al. [14], the inhibitory substances that are soluble in water can be removed with aqueous extraction, while hemicellulose and sugars can be degraded into non-inhibitory substances through alkaline hydrolysis. Then, two treatments were tested based on the results of Quiroga et al. [15]: hot water washing at 80 °C during one-hour (Hot1), and immersion in calcium hydroxide solution (concentration 1.85 g/L) during one (Alc1), two (Alc2), three (Alc3), and four (Alc4) hours. The water-to-WS ratio (in mass) used was 10. After the treatments, the WS were air dried for two days, and five WBC of WS volumetric fraction of 45% were produced with a cement matrix in order to access the best treatment. The mixture process was based on da Gloria et al. [1], and the samples obtained were submitted to a compressive test after 7 days. The compressive strength values obtained were 5.42, 4.25, 6.5, 6.45, and 5 MPa for the WBC made with Hot1, Alc1, Alc2, Alc3, and Alc4, respectively. From the result, it was concluded that 2 h of alkaline immersion is the most effective procedure to improve the compatibility of WS with cement, and thereby it was the selected treatment for the WS.

**Figure 2:**
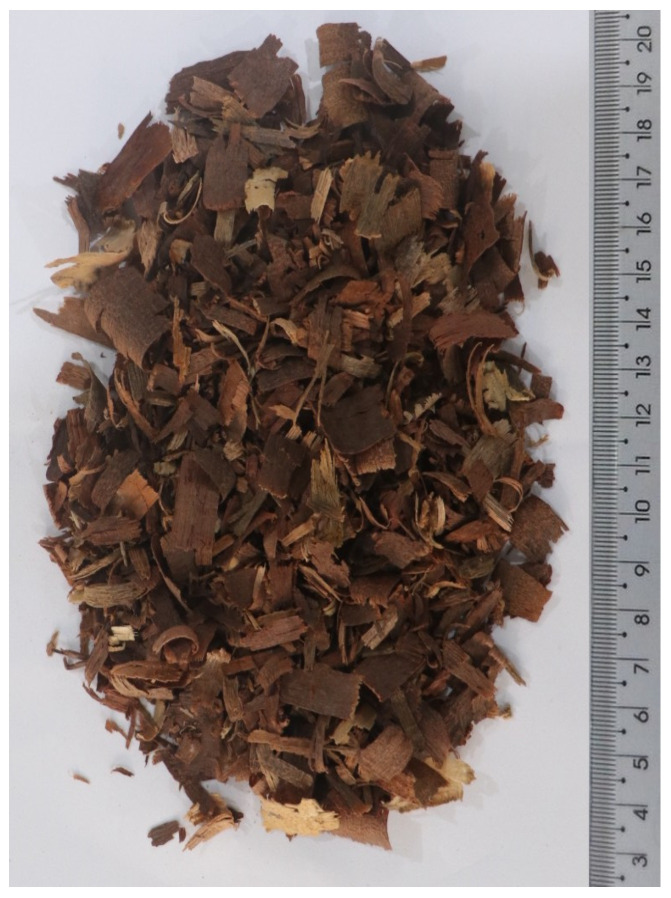
Treated wood shavings appearance.

The apparent specific gravity [16] and the total moisture content [17] of the treated WS (Figure 2) were 550 kg/m^3^ and 13.66%, respectively. The water absorption of the WS was determined according to the procedure adopted by da Gloria et al. [1]. Such procedure took into account the amount of water absorbed by the bio-aggregates during the bio-concretes production, which can be resumed as follows. A representative sample of WS (100 g) were mixed in 500 mL of water for 4 min, and after mixing, the WS were passed through a 150 μm sieve to drain the excessive water and air-dried for 5 min. Next, the WS were weighed, and the water absorption determined. After three repetitions of the test, an average of 80% of water absorption was obtained.

### 2.3. Bio-Concretes Fabrication and Testing

#### 2.3.1. Bio-Concretes Production

To develop the mix design diagram, nine WBC were produced with the volumetric fractions of 40%, 45%, and 50% of WS, and the water-to-binder ratios of 0.35, 0.40, and 0.45. The water-to-binder ratios were chosen aiming at a spreading of 200 ± 20 mm, which allowed the moulding of WBC with mechanical vibration, as recommended by Caldas et al. [9]. As the WBC is composed of WS and cementitious pastes, the WBC volume is the sum of both WS and cementitious paste volumes. The WS mass was calculated considering its apparent specific gravity and volume, while the mass of cementitious materials was deducted from the cementitious paste volume, taking into account the specific gravity of cement, metakaolin, fly ash, and the hydration water (based on the on the water-to-binder ratio). The cement mass was calculated in accordance with Equation (4) of Section 3.5.3. The total water used is a sum of the hydration water (W_H_) and the compensating water (W_C_) based on the WS water absorption. The material consumption per cubic meter is detailed in Table 2. In the nomenclature WBCX-Y and X and Y are the WS volumetric fraction and the water-to-binder ratio, respectively.

The bio-concretes were produced in a 5 L mixer, manufactured by Solotest (São Paulo, Brazil), under lab-conditions at a controlled temperature of 21 ± 1 °C. After a previous dissolution of the calcium chloride into the water, the cementitious materials and the WS were introduced into the mixer and mixed for 2 min. Next, the total water was gradually added for one minute and the mix ended after a total time of 4 min.

The consistence index of the WBC at a fresh state was measured according to the National Brazilian Standard [18]. The bio-concrete mixtures were moulded in three layers, and each layer was vibrated on a vibratory table (68 Hz) for 10 s. The bio-concretes were kept in the moulds and protected against moisture loss until demoulding after 24 h. The specimens were cured into a conditioned chamber at 20 ± 3 °C and 60% RH until 28 days of age.

#### 2.3.2. Bulk Density and Compressive Test

The bulk density and the compressive strength of the WBC were assessed by testing five cylindrical samples (diameter and height of 50 mm and 100 mm, respectively) at 28 days of age. The bulk density was determined through the ratio mass/volume of the samples, while the compressive test was performed by using the universal testing machine of model UH-F1000 kN supplied by Shimadzu (Kyoto, Japan), at the speed of 0.3 mm/min [19]. The axial deformation was monitored through two diametrically opposed linear variation displacement transformers from Controls (Liscate, Milan, Italy), and positioned over a gage length of 50 mm at the mid-height of the specimen.

### 2.4. Life Cycle Assessment (LCA) for GHG Emissions Calculation

The LCA was executed according to international standards, ISO 14040 [20], ISO 14044 [21], EN 15978:2011 [22], and EN 15804:2019 [23]. Based on the standards, the LCA was divided in the following phases: (1) Definition of Goal, Scope, and Functional Unit; (2) Life Cycle Inventory (LCI); (3) Life Cycle Impact Assessment (LCIA); and (4) Interpretation.

#### 2.4.1. Definition of Goal, Scope and Functional Unit

The goal of this LCA study is to evaluate the life cycle GHG emissions (in kgCO_2_-eq) of different mixtures of WBC. The scope, from cradle-to-gate, considers raw materials supply (A1), transport (A2), and WBC manufacturing (A3), following the recommendations of EN 15978:2011 [22] and EN 15804:2019 [23]. The Functional Unit (FU) is the volume (in m^3^) of the produced WBC.

#### 2.4.2. Life Cycle Inventory (LCI)

In the LCI phase, primary data were collected in the laboratory during WBC production and development, while secondary data were collected from the literature and Ecoinvent v. 3.8, that also considers a cradle-to-gate scope. The market transports and electricity consumption of original Ecoinvent data was adapted to the Brazilian energy mix. The data used in the modelling are described in Table 3, most of them was already developed for the context of Brazil (BR), while some data from the rest of the word (RoW) were also considered. For the transportation, three scenarios were considered in terms of transport distance of raw materials: best, intermediate, and worst, as presented in Table 4, according to Caldas et al. [9].

#### 2.4.3. Life Cycle Impact Assessment (LCIA)

For the LCIA, the EN 15804 + A2 (v. 1.00) method [25] was employed, considering the Climate Change impact: Climate Change, Fossil and Land use, and land use change. The Climate Change–Biogenic was modelled for the WS according to the method developed by Guest et al. [26] that is described in the next section.

#### 2.4.4. Biogenic Carbon Calculation

For the WS biogenic carbon (the CO_2_ sequestered by wood photosynthesis process) quantification, the method developed by Guest et al. [26] was employed, considering the approach used by Caldas et al. [27]. Such a method defines a GWPbio index, which indicates how much biogenic CO_2_ emissions contribute to climate change relative to fossil CO_2_ emissions [27]. For the GWPbio index quantification, it is necessary to know the time (in years) of the biogenic CO_2_ storage period in the anthroposphere and the biomass rotation period (in years). The rotation period of biomass refers to the duration or frequency at which biomass resources are harvested or renewed in forestry and agriculture practices. The method was chosen because it considers the influence of time in GHG emissions impact, which tends to bring more reliable results [28]. It was considered that the biologic CO_2_ is stored indefinitely (for more than 100 years) since the cementitious materials of bio-concretes tend to retain the biomass [29]. The calculation of the biogenic amount of wood shaving was adjusted for the moisture content already present in the biomass (13.66%). Furthermore, based on the rotation period of 10 year of the Pinus and Eucalyptus grown [9,28] and the storage period in the anthroposphere of 100 years, the GWPbio factor of −96% was found. The minus signal for this factor indicates that this WBC has the potential to generate carbon credits. For the sensitivity analysis, different values of biogenic carbon are considered, representing their scenarios (best, intermediate, and worst), and these values are presented in Table 5. It is worth noting that if the amount of carbon in dry matter (C%) is higher, more CO_2_ will remain stored in the WBC, resulting in less GHG emissions.

#### 2.4.5. GHG Emissions-Mechanical Performance Intensity Indicator

A GHG emissions-mechanical performance indicator (in kgCO_2_-eq/m^3^.MPa) for GHG emissions was adopted to verify the differences between the compressive strength of the WBC and carbon, like the indicator used by Celik et al. [30] and Caldas et al. [9]. Only positive GHG emissions are accounted for this evaluation since negative values will not make sense.

## 3. Results and Discussions

### 3.1. Workability

**Figure 3:**
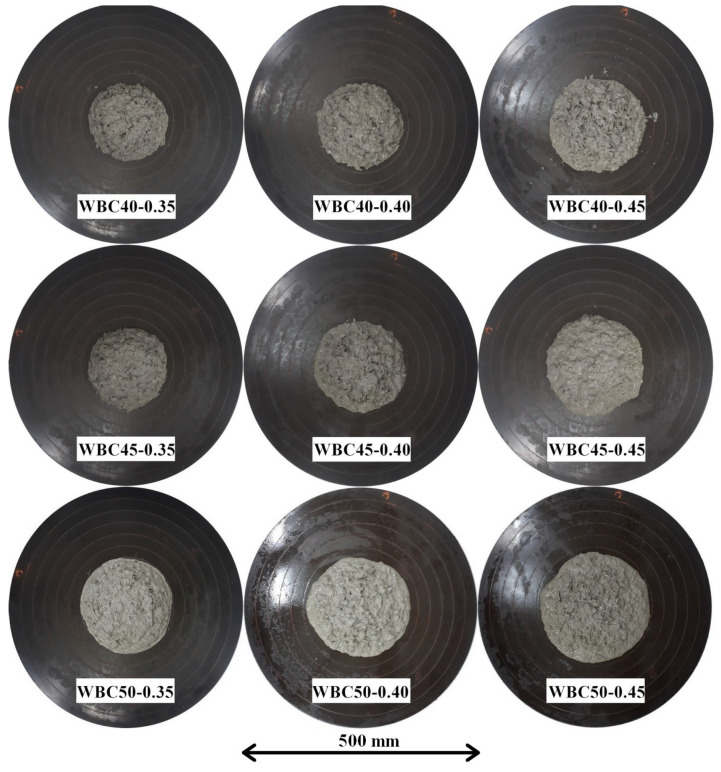
Spreading of the WBC.

The WBC consistence indexes are listed in Table 6, while their spreading is shown in Figure 3.

The results showed that through the mixing procedure, it was possible to achieve workable WBC without exudation and segregation. The obtained indexes ranged from 170 to 240 mm. As expected, the spreading increased with the w/b ratio. It was also observed that the higher the volumetric fraction, the higher the index. Although this behaviour is not a priori expected, it can be justified by analysing the total water (sum of hydration and compensating water)-to-binder ratio. Such a ratio was revealed to be higher for higher WS content due to the addition of compensating water.

### 3.2. Bulk Density

The bulk density values of the WBC are detailed in Table 7. According to the results, the density varied from 715 to 1207 kg/m^3^, indicating that the WBC produced can be classify as lightweight materials according to the RILEM functional classification. As expected, it was observed that the density decreases with the increase in both the WS volume and water-to-binder ratio.

Comparing the results of Table 6 and Table 7, it can be observed that the higher the WBC spreading, the lower the density. This trend can be explained by the influence of the free water in the mixture. At a fresh state, a high volume of free water promoted a higher spreading of the mixture, probably due to an air content increase. The air content will promote higher porosity at a hardened state and, consequently, lower density.

### 3.3. Compressive Strength

**Figure 4:**
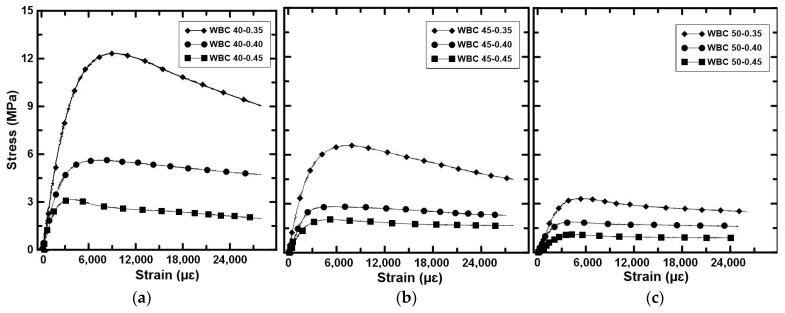
Compressive stress–strain curves: (**a**) WBC40, (**b**) WBC45, (**c**) WBC50.

The compressive strength and Young’s modulus of the WBC after 28 days are summarised in Table 8, while the stress–strain curves are illustrated in Figure 4.

The stress–strain curves showed an initial linear behaviour up to approximately 60% of the compressive strength, followed by a non-linear phase until reaching the peak stress. Then, a softening branch, characterized by a slight load decrease (around 10%) is observed until 25,000 με, except in WBC40-0.35 and WBC45-0.35, which presented load decrease of 27% and 34%, respectively. WBC40-0.35 presented strength two and almost four times higher than WBC40-0.40 and WBC40-0.45, respectively. The increase of 0.05 of the w/b ratio induced, in average, a strength reduction of 48% within the WBC40. When compared to WBC45-0.40 and WBC45-0.45, WBC45-0.35 presented strength 60% and 70% higher, respectively. The WBC45 showed around 57% of the WBC40 strength for the same w/b ratio. Finally, the WBC50 presented the lowest strength. WBC50-0.35 strength was 43% and 66% higher than those of WBC50-0.40 and WBC50-0.45, respectively.

The Young’s modulus followed the same trend: the higher the w/b ratio or volume of WS, the lower the Young’s modulus. The porosity of the bio-concretes increased with the WS and water content, which directly decreased both strength and stiffness.

### 3.4. GHG Emissions

**Figure 5:**
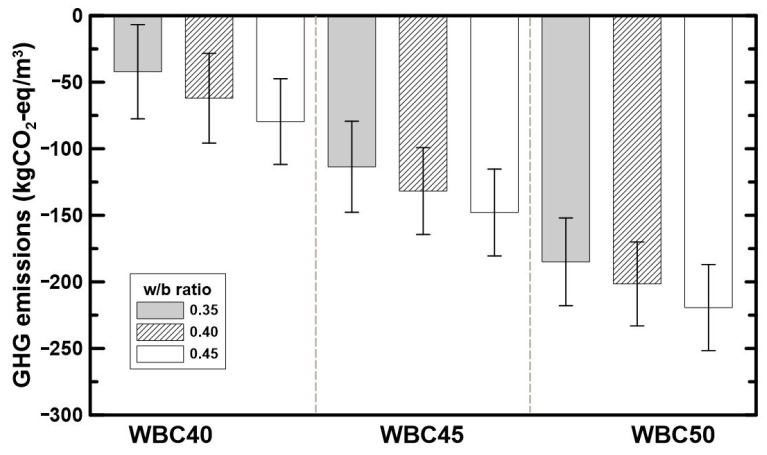
Life cycle GHG emissions of the evaluated WBC.

The GHG emissions calculated based on the experimental results of each WBC are presented in Table 9 and Figure 5.

It can be observed that average values of GHG emissions of all mixtures are negative, even for worst scenarios (top error bars) that consider higher transport distances and less biogenic carbon. In other words, all evaluated WBC can generate carbon credits, since more carbon is stocked than released in the atmosphere, confirming the potential of bio-concretes as presented by Caldas et al. [27]. It is also noted that an expressive reduction in GHG emissions due to the decrease in CEM and the increase in WS, reaching a diminution of 80% between WBC40-0.35 and WBC50-0.45.

It is worth mentioning that the alkaline treatment did not have a significant impact on the environmental balance because of the few amount of calcium chloride used per WS mass. This treatment was a good replacement for the hot water washing that demands a lot of energy to heat the water before introducing the bio-aggregates.

**Figure 6:**
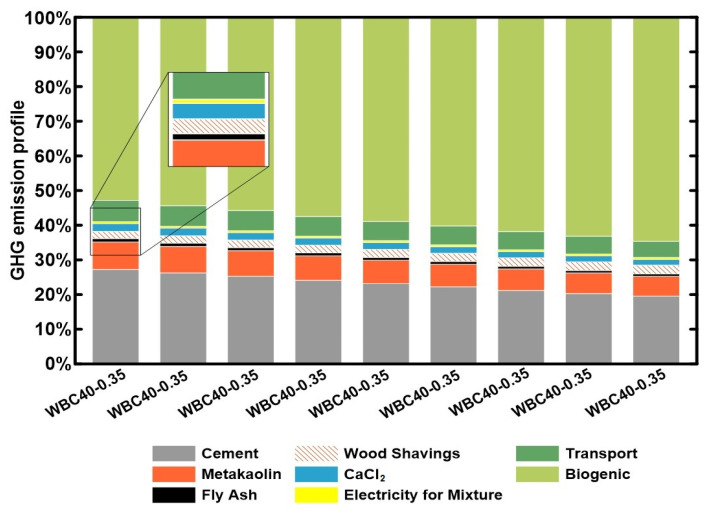
GHG emissions profile of the evaluated WBC.

Finally, in Figure 6, the influence of different raw materials and processes in the GHG emissions profile, considering the biogenic carbon (the CO_2_ sequestered by photosynthesis process), of WBC can be evaluated and understood.

**Figure 7:**
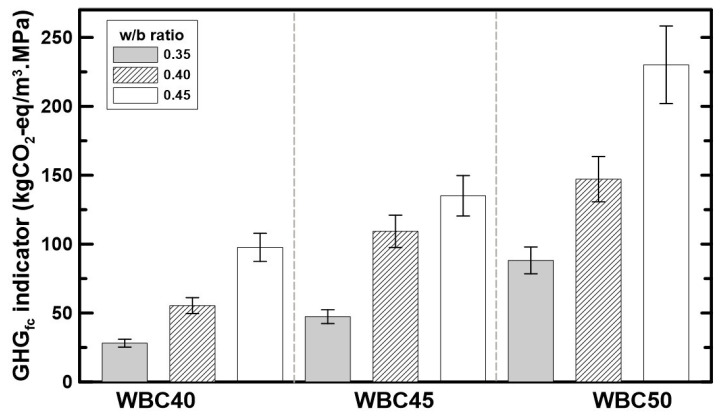
GHG emissions-mechanical performance indicator.

The main binders, CEM and MK, have a significant influence, since they emit a great amount of GHG emissions during their production process, especially due to the calcination process that liberates high amounts of CO_2_. The use of FA, a waste-based SCM, is also a good strategy and is frequently used for the reduction in carbon footprint of concretes [30]. The biogenic carbon in WS is the most influential factor in the WBC life cycle’s GHG emissions, showing that the increase in its content in WBC mixture is the most efficient strategy to reduce GHG emissions. These findings agree with the previous study [27]. Nonetheless, none of the previous evaluated mixtures reached such low GHG emissions results for WBC. On the other hand, the WBC studied here stayed in the same level of hempcretes that are normally the bio-concretes with the smallest GHG emissions available [27]. The increase in WS amount led to a decrease in the density and compressive strength of WBC, as presented before. Therefore, it is necessary to have a clear definition of the desired application of WBC in order to have adequate mechanical performance with the smallest amount of GHG emissions. For that reason, the GHG_fc_ indicator, which represents the combined concept of GHG and compressive strength of the material, was calculated. When the GHG_fc_ indicator is evaluated (in Figure 7), it can be observed that the mixtures with 40% of aggregates in volume (WBC 40) and with less water content are more efficient in terms of GHG emissions (less emissions) for each gain of 1 MPa. Therefore, considering structural and environmental exigencies, these are the recommended mixtures.

### 3.5. Mix Design Diagram Construction

Based on the experimental results obtained, two mix design diagrams are proposed. Such diagrams are based on the relationships between the WS volumetric fraction, the compressive strength at 28 days, the bulk density, the water-to-binder ratio, the cement content, and the GHG emissions.

#### 3.5.1. Compressive Strength vs. Bulk Density

**Figure 8:**
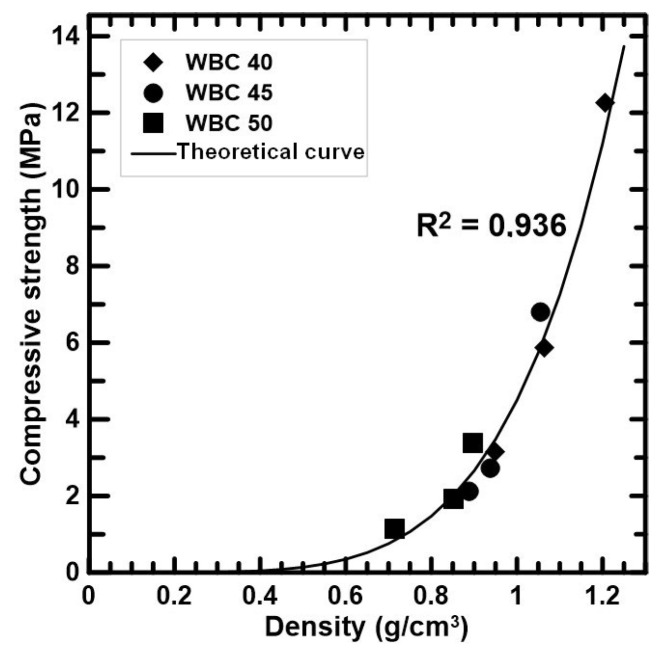
Relationship between density and compressive strength.

From the experimental results, the compressive strength at 28 days (f_c28_) and the WBC density (γ) can be correlated through the polynomial Equation (1).

f_c28_ = k_1_·γ^5^ (1)

where k_1_ = 4.5 was obtained from calibration process. Figure 8 shows the experimental and theoretical results of the compressive strength in function of the density. According to the graph and the R^2^ coefficient, the theoretical curve predicts the experimental values with high accuracy.

#### 3.5.2. Compressive Strength vs. Water-to-Binder Ratio

Two equations were determined to predict the compressive strength at 28 days (f_c28_) from the w/b ratio. One of them is based on Abrams’ law:

f_c28_ = k_2_/k_3_^(w/b)^ (2)

where k_2_ is a constant calibrated for WBC of same volumetric fraction V_f_ (k_2_ = 20,000V_f_^2^ − 25,000V_f_ + 7800) and k_3_ = 3.2 × 10^5^ for all WBC. For WBC40, WBC45, and WBC50, k_2_ values are 1000, 600, and 300, respectively.

**Figure 9:**
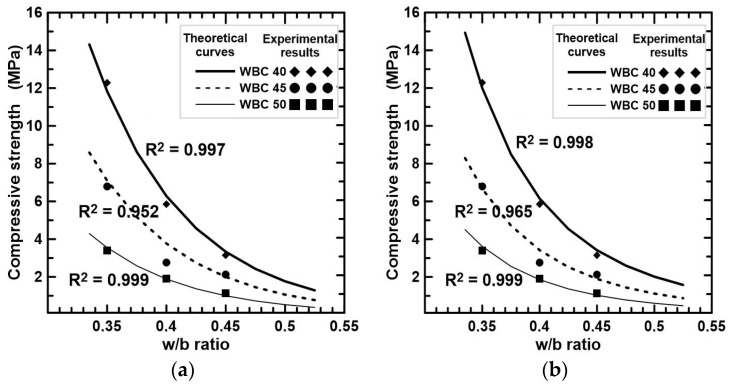
Relationship between compressive strength and w/b ratio. (**a**) Equation (2); (**b**) Equation (3).

The second approach is based on the following equation:

f_c28_ = k_4_/(w/b)^k^_5_ (3)

where k_4_ is calibrated for WBC of the same V_f_ (k_4_ = 2.4V_f_^2^ − 2.6V_f_ +0.719), and k_5_ = 5 was obtained for all WBC. The values of k_4_ for WBC40, WBC45, and WBC50 are, respectively, 0.063, 0.035, and 0.019. Figure 9 compares f_c28_ versus w/b registered experimentally and those obtained from Equations (2) and (3). The R^2^ coefficients demonstrate that both equations predict, with high accuracy, the f_c28_ for the considered interval of w/b. The minimum predictive performance was registered in the case of WBC45 mixtures due to the overestimation of f_c28_ for the series with w/b = 0.40.

#### 3.5.3. Cement Content vs. Water-to-Binder Ratio

The cement content of the WBC presented in Table 2 was calculated according to the following Equation (4):

(4) $$C=V\times \frac{1-V_{f}}{\frac{1}{\rho_{c}}+\frac{w/b}{0.3\times \rho_{w}}+\frac{1}{\rho_{MK}}+\frac{0.4}{0.3\times \rho_{FA}}}$$

where C is the cement consumption (kg), V the mixture volume (m^3^), V_f_ the WS volumetric fraction (%), w/b the water-to-binder ratio, ρ_c_ the cement density (kg/m^3^), ρ_w_ the water density (kg/m^3^), ρ_MK_ the MK density (kg/m^3^), and ρ_FA_ the FA density (kg/m^3^).

When the water-to-binder ratio is between 0.35 and 0.50, a linear tendency between cement content and w/b is obtained. Therefore, Equation (4) can be simplified as:

C = k_6_ − k_7_ × (w/b) (5)

where k_6_ and k_7_ are coefficients calibrated for WBC of different V_f_ of WS, whose values are indicated in Table 10.

**Figure 10:**
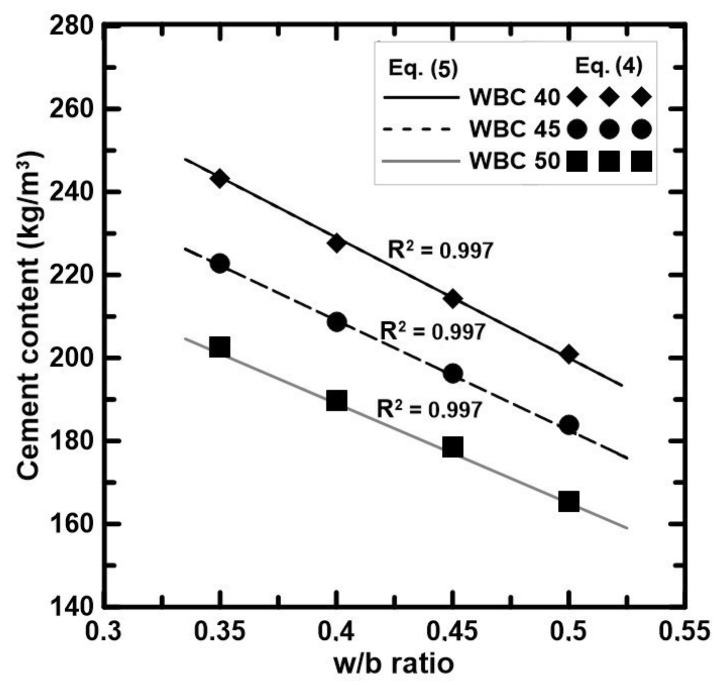
Linear relationship between cement content and w/b ratio.

The cement content per cubic meter obtained from Equation (4) for w/b = 0.35, 0.40, 0.45, 0.50 and Equation (5) for the interval 0.32 < w/b < 0.52 are compared in Figure 10, which highlights the linear relationship between cement content and the w/b ratio.

#### 3.5.4. Cement Content vs. GHG Emissions

When the cement content of WBC40, WBC45, and WBC50 is, respectively, between 190 and 250, 175 and 230, and 160 and 210 kg/m^3^, a linear tendency between cement content and the GHG emissions is observed. The relationship can be expressed as follows:

GHG_em_ = k_8_ − k_9_×C (6)

where k_8_ and k_9_ are coefficients calibrated for WBC of different V_f_ of WS, whose values are indicated in Table 11.

**Figure 11:**
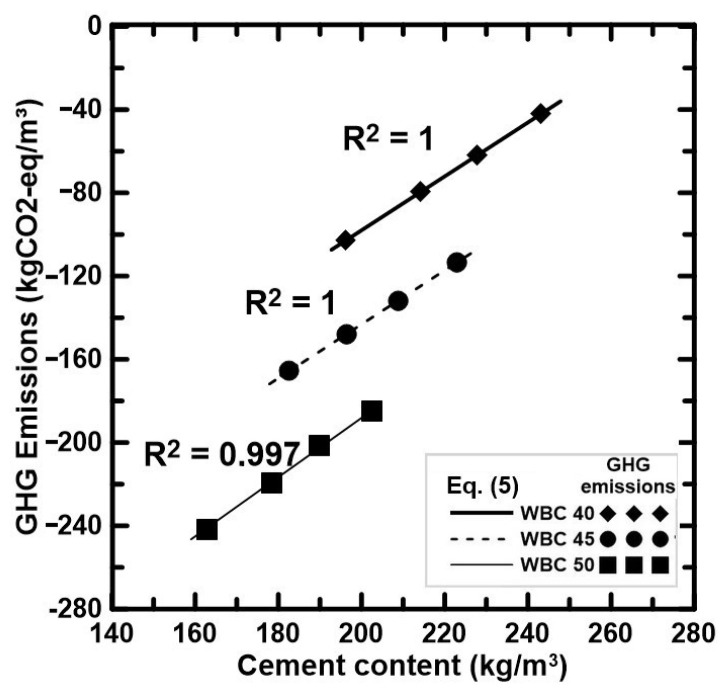
Linear relationship between cement content and GHG emissions.

Figure 11 highlights the linear relationship between cement content and GHG emissions.

#### 3.5.5. Mix Design Diagrams Construction

**Figure 12:**
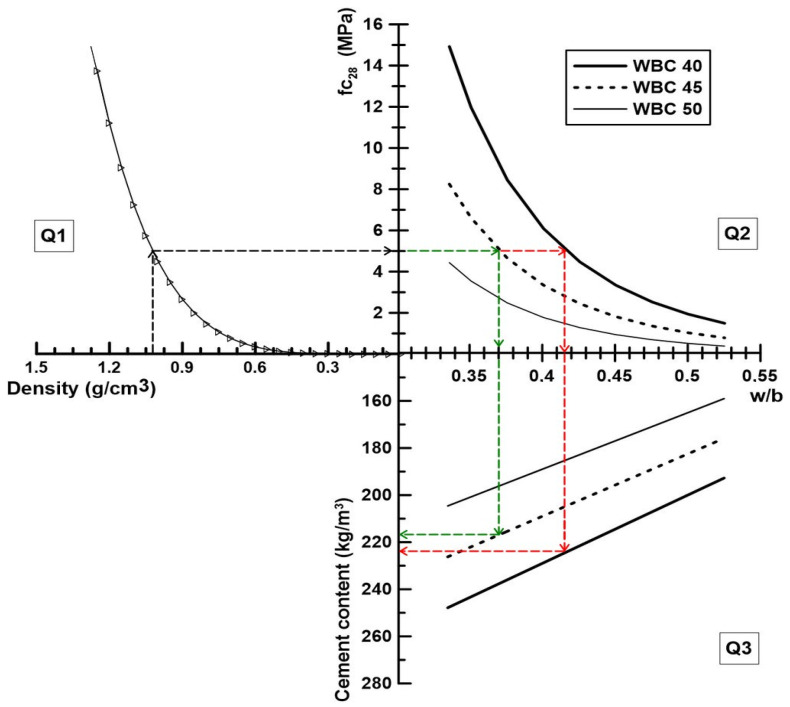
Mix design diagram of WBC starting from the density.

Based on the equations previously obtained, two WBC diagrams are proposed to design the WBC. In the first diagram, the WBC can be determined to attain a target compressive strength or a bulk density, while in the second one, the WBC is designed to achieve an aimed compressive strength and GHG emissions. The first diagram (Figure 12) is divided into the following three quadrants:

- Quadrant 1 (Q1): Select a target density and determine the corresponding compressive strength.
- Quadrant 2 (Q2): From the compressive strength, choose a V_f_ and determine the w/b ratio.
- Quadrant 3 (Q3): Determine the cement content for the adopted WBC V_f_ and w/b ratio.

Figure 12 presents the successive steps for designing two WBCs, starting from the target density (vertical black dash line in Q1). To obtain a WBC of 1.02 g/cm^3^, the projection on the curve of Q1 shows that the equivalent compressive strength is 5 MPa. That strength can be reached with two V_f_ of WS, 45% and 40%, according to the curves of Q2. Supposing that 45% is the chosen V_f_ (horizontal green dash-line), the projection from the curve to the w/b axis indicate w/b = 0.37. On Q3, once the value of 0.37 is projected successively on the curve WBC45 and the cement content axis, it can be obtained a cement content of 217 kg/m^3^. As the binder was composed, in mass, of 30% of cement, 30% of MK and 40% of FA, the FA and MK content are 217 and 288 kg/m^3^, respectively. When V_f_ = 40% (red dash), w/b = 0.415, C = MK = 224 kg/m^3^ and FA = 299 kg/m^3^.

**Figure 13:**
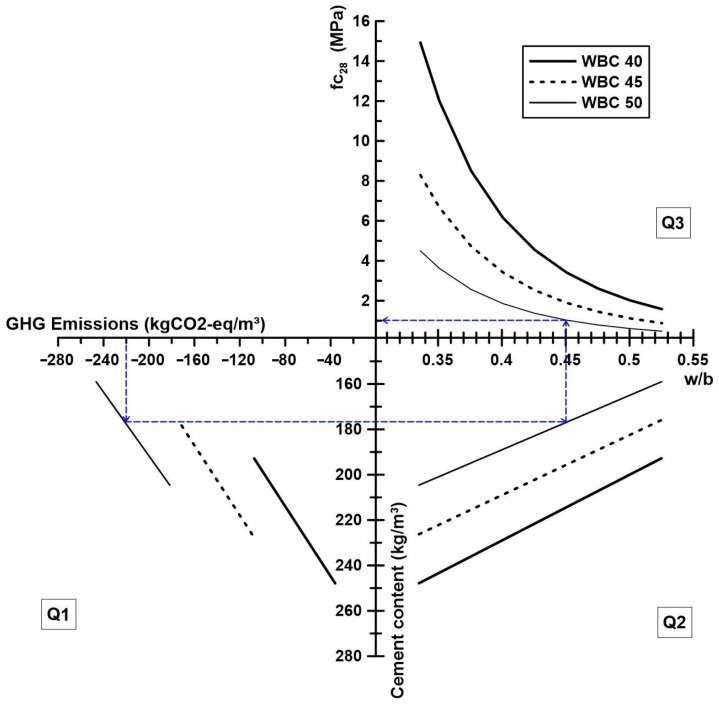
Mix design diagram of WBC starting from the GHG emissions.

The second diagram (Figure 13) is divided in the following three quadrants:

- Quadrant 1 (Q1): Select a target GHG emissions value, the corresponding V_f_ and determine the cement content.
- Quadrant 2 (Q2): Determine the w/b ratio.
- Quadrant 3 (Q3): Determine the compressive strength.

Figure 13 presents the steps for designing a WBC, starting from the target of the GHG emissions (blue dash). To produce a WBC of GHG = −220 kgCO_2_-eq/m^3^ (blue dash), the equivalent V_f_, cement content, and w/b ratio are 50%, 177 kg/m^3^, and 0.45, respectively. From that mixture, it is expected to obtain a compressive strength of 1 MPa at 28 days of age.

## 4. Conclusions

This paper describes the production and characterization of wood bio-concretes (WBC) with low cement content, and proposes a rational mix design approach that considers the greenhouse gas (GHG) emissions. Based on the results obtained, it was possible to conclude that:

- The alkaline treatment of the wood shavings was efficient in terms of improving the chemical compatibility between cement and wood. Such a positive result allowed us to treat the WS without using heat energy, and also reduced the amount of water over the procedure that used several hot washing cycles.
- The combination of metakaolin and fly ash lead to a 70% of cement substitution, which promoted the development of workable low-carbon WBC, with negative GHG emissions (or in other words, with potential to generate carbon credits).
- The increase in wood shavings, metakaolin, and fly ash content significantly decreased the life cycle GHG emissions, reaching a reduction of 80%.
- In a hardened state, the bio-concretes presented compressive strength varying between 1.15 and 12.27 MPa. The bio-concretes with low cement content demonstrated a high-strength decrease when the water-to-binder ratio was increased. This behaviour was also demonstrated by GHG’s emissions-mechanical performance (in kgCO_2_-eq/m^3^. MPa). In other words, for the gain of 1 MPa of resistance, the mixtures with more water content will emit more GHG emissions.
- From the experimental results, a mix design diagram was developed. This diagram can satisfactorily estimate the compressive strength of WBC with 40%, 45%, and 50% of wood, produced by setting the water-to-binder ratio between 0.33 and 0.52.
- This diagram includes a quadrant dedicated to the GHG emissions, being also a parameter considered in the mix design diagram of WBC. It can be used for other bio-concretes and can be a helpful way to evaluate and archive low-carbon targets during mix designs.
- The diagram confirmed the expectation in a previous publication regarding the possibility of the dosage method extension to other types of bio-concretes.
- The diagram obtained can be used for designing bio-concretes containing other types of bio-aggregates once their volumetric fractions are the same as used in the present paper, and also maintaining the proportion of cementitious materials.

## Figures and Tables

**Table 1:** Chemical properties and density of the cementitious materials.

Chemical Components	CEM (%)	MK (%)	FA (%)
CaO	68.973	-	1.948
SiO_2_	14.955	50.949	52.246
Al_2_O_3_	4.701	42.224	33.806
Fe_2_O_3_	3.506	1.982	4.910
K_2_O	0.988	1.983	3.445
SO_3_	4.296	1.202	1.793
SrO	0.425	0.004	0.024
MnO	0.140	0.009	0.039
ZnO	0.014	0.007	0.041
Density (kg/m^3^)	3170	2810	2160

**Table 2:** Materials consumption in kg/m^3^ of wood bio-concretes.

Bio-Concretes	WS	CEM	MK	FA	W_H_	W_C_	CaCl_2_
WBC40-0.35	220.0	243.13	243.13	324.17	283.65	176.0	24.31
WBC40-0.40	220.0	227.75	227.75	303.67	303.67	176.0	22.77
WBC40-0.45	220.0	214.20	214.20	285.60	321.30	176.0	21.42
WBC45-0.35	247.5	222.87	222.87	297.16	260.01	198.0	22.29
WBC45-0.40	247.5	208.77	208.77	278.36	278.36	198.0	20.88
WBC45-0.45	247.5	196.35	196.35	261.80	294.52	198.0	19.63
WBC50-0.35	275.0	202.61	202.61	270.15	236.38	220.0	20.26
WBC50-0.40	275.0	189.79	189.79	253.05	253.05	220.0	18.98
WBC50-0.45	275.0	178.50	178.50	238.00	267.75	220.0	17.85

**Table 3:** Raw materials, activities and datasets used in wood bio-concrete (WBC) life cycle inventory (LCI).

Materials and Activities	Dataset	GHG Factor
Wood Shavings (WS)	Shavings, MIXwood, measured as dry mass {RoW}| suction, shavings	0.07 kgCO_2_-eq/kg
Cement (CEM)	Cement, Portland {BR}| cement production, Portland	0.82 kgCO_2_-eq/kg
Metakaolin (MK)	Calcined clay {BR}| calcined clay production	0.24 kgCO_2_-eq/kg
Fly Ash (FA)	Modelled by the authors based on Chen et al. [24] and electricity, medium voltage {BR}	0.21 kgCO_2_-eq/kg
Calcium Chloride (CaCl_2_)	Calcium chloride {RoW}| soda production, solvay process	0.68 kgCO_2_-eq/kg
Calcium Hydroxide (Ca(OH)_2_)	Lime-hydrated-packed {RoW}| production	0.94 kgCO_2_-eq/kg
Water (W_w_^1^, W_H_ and W_C_)	Tap water {BR}	0.001 kgCO_2_-eq/kg
Transportation	Transport, freight, lorry 16–32 metric ton, EURO3 {BR}	0.13 kgCO_2_-eq/t.km
Electricity	Electricity, medium voltage {BR}| market group for electricity	0.19 kgCO_2_-eq/kWh
WBC production	Concrete, 25 MPa {BR}| concrete production ^2^	4.21 kgCO_2_-eq/m^3^

^1^ W_w_ wood shavings washing water. ^2^ It was considered just the processes that occur inside the concrete plant (diesel, electricity, water, and lubricating oil consumption).

**Table 4:** Transport distance (km) of raw materials for different scenarios.

Scenarios	WS	CEM	MK	FA	CaCl_2_
Best	100	50	50	100	200
Intermediate	200	100	200	200	400
Worst	800	400	800	800	1000

**Table 5:** Parameters and data for the biogenic carbon modelling of wood shavings.

Scenarios	C (%)	Time in Anthroposphere (Years)	GWPbio Factor (%)	Biogenic Carbon (kgCO_2_/kg)
Best	53	100	−96	−1.86
Intermediate	50	100	−96	−1.76
Worst	47	100	−96	−1.65

**Table 6:** Consistency indexes (mm) of the WBC.

	w/b 0.35	w/b 0.40	w/b 0.45
WBC40	170	185	210
WBC45	180	190	210
WBC50	200	220	240

**Table 7:** Apparent density (kg/m^3^) of the WBC (Standard deviation in brackets).

	w/b 0.35	w/b 0.40	w/b 0.45
WBC40	1206.83 (±4.7)	1063.45 (±7.2)	967.95 (±9.6)
WBC45	1053.83 (±3.1)	938.38 (±4.7)	886.59 (±11.7)
WBC50	895.51 (±12.8)	853.33 (±15.2)	715.15 (±12.3)

**Table 8:** Compressive strength and Young modulus of the WBC (Standard deviation in brackets).

Bio-Concretes	Compressive Strength (MPa)	Young Modulus (GPa)
WBC40-0.35	12.27 (±0.3)	3.31 (±0.2)
WBC40-0.40	5.87 (±0.1)	2.23 (±0.2)
WBC40-0.45	3.15 (±0.2)	1.62 (±0.1)
WBC45-0.35	6.80 (±0.3)	2.44 (±0.1)
WBC45-0.40	2.78 (±0.1)	1.28 (±0.1)
WBC45-0.45	2.13 (±0.0)	1.01 (±0.1)
WBC50-0.35	3.39 (±0.1)	1.43 (±0.1)
WBC50-0.40	1.92 (±0.1)	1.06 (±0.1)
WBC50-0.45	1.15 (±0.0)	0.64 (±0.1)

**Table 9:** Average values of the WBC GHG emissions (Standard deviation in brackets).

Bio-Concretes	GHG Emissions (kgCO_2_-eq/m^3^)
WBC40-0.35	−42.11 (±35.42)
WBC40-0.40	−62.04 (±33.70)
WBC40-0.45	−79.59 (±32.19)
WBC45-0.35	−113.52 (±34.19)
WBC45-0.40	−131.79 (±32.61)
WBC45-0.45	−147.89 (±31.23)
WBC50-0.35	−184.94 (±32.96)
WBC50-0.40	−201.55 (±31.52)
WBC50-0.45	−219.32 (±32.36)

**Table 10:** k_6_ and k_7_ values.

Bio-Concretes	k_6_	k_7_
WBC40	345	290
WBC45	315	265
WBC50	285	240

**Table 11:** k_8_ and k_9_ values.

Bio-Concretes	k8	k9
WBC40	1.2956	357.10
WBC45	1.2959	402.33
WBC50	1.4232	472.78

## Data Availability

The original contributions presented in the study are included in the article, further inquiries can be directed to the corresponding author.
